# Prevalence and clinical characteristics of hypertension and metabolic syndrome in newly diagnosed patients with ketosis-onset diabetes: a cross-sectional study

**DOI:** 10.1186/s13098-019-0426-x

**Published:** 2019-04-25

**Authors:** Jun-Wei Wang, Ai-Ping Wang, Ming-Yun Chen, Jun-Xi Lu, Jiang-Feng Ke, Lian-Xi Li, Wei-Ping Jia

**Affiliations:** 1 Department of Endocrinology and Metabolism, Shanghai Clinical Center for Diabetes, Shanghai Diabetes Institute, Shanghai Key Laboratory of Diabetes Mellitus, Shanghai Key Clinical Center for Metabolic Disease, Shanghai Jiao Tong University Affiliated Sixth People’s Hospital, 600 Yishan Road, Shanghai, 200233 China; 2 Department of Endocrinology, 454 Hospital of PLA, Nanjing, 210002 Jiangsu Province China

**Keywords:** Hypertension, Ketosis-onset diabetes, Metabolic syndrome

## Abstract

**Background:**

To investigate the prevalence and clinical characteristics of hypertension (HTN) and metabolic syndrome (MetS) in newly diagnosed diabetes with ketosis-onset.

**Methods:**

A cross-sectional study was adopted in 734 newly diagnosed diabetics including 83 type 1 diabetics with positive islet-associated autoantibodies, 279 ketosis-onset diabetics without islet-associated autoantibodies and 372 non-ketotic type 2 diabetics. The clinical characteristics of HTN and MetS were compared across the three groups, and the risk factors of them were appraised in each group.

**Results:**

The prevalence of HTN and MetS were substantially higher in the ketosis-onset diabetics (34.4% for HTN and 58.8% for MetS) than in the type 1 diabetics (15.7% for HTN, *P *= 0.004; 25.3% for MetS, *P *< 0.001), but showed no remarkable difference compared with the type 2 diabetics (42.7% for HTN, *P *= 0.496; 72.3% for MetS, *P *= 0.079). Furthermore, the risk factors for both HTN and MetS in the ketosis-onset diabetics resembled those in the type 2 diabetics, but significantly different from those in the type 1 diabetics.

**Conclusions:**

The prevalence of HTN and MetS in the ketosis-onset diabetics were magnificently higher than in the type 1 diabetics but showed no difference in comparison to the type 2 diabetics. Likewise, the clinical features and risk factors of HTN and MetS in the ketosis-onset diabetes resembled those in the type 2 diabetes but differed from those in the type 1 diabetes. Our findings indicate that ketosis-onset diabetes should be classified into type 2 diabetes rather than idiopathic type 1 diabetes.

## Background

Ketosis-onset diabetes or ketosis-prone diabetes, once termed atypical diabetes, has been categorized as idiopathic type 1 diabetes based on the American Diabetes Association [1–3]. However, several studies suggested that ketosis-onset diabetes was characterized as higher age of onset, overweight, the family history of diabetes, discontinuation of insulin treatment after euglycemic remission, and a variable response to diet and oral hypoglycemic agents, which supported that ketosis-onset diabetes was more likely to be a sub-group of type 2 diabetes [2, 4].

The immunologic and metabolic characteristics of ketosis-onset diabetes have been well established, such as lack of islet-related antibodies and human leukocyte antigen (HLA) genetic association, unprovoked ketoacidosis, a remarkable but transient defect in insulin secretion, near-normoglycemic remission, substantial beta-cell reserve and displayed muscle, adipose tissue and hepar tissue insulin resistance [2, 5–7]. Similar to type 2 diabetics, patients with ketosis-onset diabetes were observed with low frequencies of type 1 diabetic susceptibility or resistance alleles [7–9]. Mauvais-Jarvis et al. [1] reported that insulin secretory capacity in ketosis-onset diabetes was lost slowly, approaching that in type 2 diabetes, whereas differing from that in type 1 diabetes. However, the clinical manifestations of ketosis-onset diabetes were mixed with those of both type 1 and non-ketotic type 2 diabetes, which reflected the difficulty of classifying this heterogeneous group. Our previous studies investigated the carotid and femoral atherosclerosis in ketosis-onset diabetes and found that ketosis-onset diabetes resembled non-ketotic type 2 diabetes in features of atherosclerotic lesions [10, 11]. We also found that the frequency and risk factors of non-alcoholic fatty liver disease in ketosis-onset diabetes were close to those in non-ketotic type 2 diabetes rather than those in type 1 diabetes [12].

HTN constituted one of the most common complications in patients with type 2 diabetes [13], which doubled the risk of cardiovascular events and deaths compared with subjects with normal blood pressure [14]. HTN was presented in over 50% of patients with type 2 diabetes [15]. For example, Coats et al. [13] reported that up to 80% of type 2 diabetes was observed to meet the HTN criteria. Furthermore, Sowers et al. [14] indicated that the prevalence of HTN in patients with type 2 diabetes was about three times higher than in patients without diabetes.

In contrast, a lower prevalence of HTN was presented in type 1 diabetes versus type 2 diabetes. The American Diabetes Association and the European Association for the Study of Diabetes reported less than one-third of type 1 diabetic patients complicated with HTN [16]. In EURODIAB study, the percentage of HTN was 24% in type 1 diabetes [17]. However, the frequency and clinical characteristics of HTN in ketosis-onset diabetes remained unclear. Tan et al. [18] evinced difference of both systolic and diastolic blood pressure among non-ketotic type 2, type 1, and ketosis-onset diabetes. However, the levels of blood pressure were observed to be comparable between ketosis-onset and non-ketotic type 2 diabetes in other studies [19–21].

Furthermore, metabolic syndrome (MetS), comprised of impaired glucose regulation, HTN, dyslipidemia, and central obesity, existed in approximately two-thirds of type 2 diabetic patients or more [22]. Our previous reports also found more MetS in type 2 diabetes [23]. Both the PROSPER study and BRHS study indicated that MetS and its components were related to type 2 diabetes [24]. O’Neil et al. [25] mentioned that MetS increased the risk of type 2 diabetes fivefold. Also, type 2 diabetes was in relation to incremental levels of serum proinflammatory cytokines and non-esterified fatty acids, and reduced glucose disposal, which contributed to the development of insulin resistance, sympathetic activation, and MetS, but such inflammatory changes were mild or nonexistent in type 1 diabetes [22, 26]. MetS was not originally introduced to identify the traits of type 1 diabetes. A previous review summarized that 8–40% type 1 diabetes were combined with MetS, lower than that in type 2 diabetes [16]. The emergence of MetS in type 1 diabetes might be attributed to advanced age, duration of diabetes, renal function, and insulin therapy, all of which were independently relevant to MetS [27]. However, Lontchi-Yimagou et al. [7] mentioned that inflammatory responses between ketosis-onset diabetes and type 2 diabetes remained similar. Furthermore, the prevalence of MetS in ketosis-onset diabetes was seldom investigated, only with the description of some components of MetS.

Therefore, our primary aims are to estimate the prevalence of HTN and MetS in ketosis-onset diabetes, and to comparatively study the clinical characteristics and risk factors of HTN and MetS across type 1, ketosis-onset and non-ketotic type 2 diabetes.

## Methods

### Study population

The cross-sectional study consecutively enrolled Chinese newly diagnosed diabetic patients aged greater than or equal to 17 years in our department from June 2007 to June 2009. The ethics review boards of Shanghai Jiao Tong University Affiliated Sixth People’s Hospital approved this survey and each patient gave written informed consent. The study accorded with the Declaration of Helsinki. Data for this report were partly derived from our previous research [10–12]. The inclusion and exclusion criteria for this study were consistent with our previous studies [10–12]. Briefly, the inclusion criteria were: age ≥ 17 years old; without history of diabetes; positive urine ketones when diabetes was diagnosed; determination of islet-associated autoantibodies. The exclusion criteria were: with concomitant conditions that might cause positive urine ketosis such as renal insufficiency, corticoid therapy, and severe infection; gestational diabetes; without complete clinical data. After exclusion of patients failed to meet inclusion terms or had incomplete information, the remaining 734 patients were available and divided into three categories by diagnostic criteria described previously [10–12]. 83 subjects were identified as type 1 diabetes with newly diagnosed diabetes accompanied with glutamic acid decarboxylase (GAD) and/or tyrosine phosphatase-like islet antigen 2 (IA-2) autoantibodies, 279 subjects as ketosis-onset diabetes with newly diagnosed diabetes and diabetic ketosis negative for both GAD and IA-2 autoantibodies, and 372 subjects as non-ketotic type 2 diabetes with newly diagnosed diabetes in the absence of islet-related autoantibodies.

### Examination and laboratory measurements

Weight, height, waist circumference, hip circumference, and blood pressure were conducted for individuals by the prior protocols [10–12, 23, 28]. Body mass index (BMI) was measured as the ratio of the weight to the height squared. Waist-to-hip ratio (WHR) was counted as the waist circumference over the hip circumference.

Laboratory measurements including fasting plasma glucose (FPG), 2 h postprandial plasma glucose (2-h PPG), fasting C-peptide (FCP), 2 h postprandial C-peptide (2-h PCP), glycosylated hemoglobin A1C (HbA1C), total cholesterol (TC), triglycerides (TG), high-density lipoprotein cholesterol (HDL-C), low-density lipoprotein cholesterol (LDL-C), alanine transaminase (ALT), serum creatinine (SCr), serum uric acid (SUA), and C-reactive protein (CRP) were determined by standard laboratory protocols [10–12, 23, 28]. The homeostasis model assessment of insulin sensitivity (HOMA2-%S) and HOMA of insulin resistance (HOMA2-IR) were separately calculated by the validated HOMA2 calculator (accessed at http://www.dtu.ox.ac.uk). Islet-related autoantibodies to GAD and IA-2 were determined by ELISA, and urine ketones were evaluated by Legal’s test. The 24 h urinary albumin excretion (UAE) was possessed as the mean value of three 24-h UAE after admission. The estimated glomerular filtration rate (eGFR) was calculated by the equation for Chinese individuals: 175 × (serum creatinine)^−1.234^ × (age)^−0.179^ (× 0.79 if female) [29].

### Definition of HTN and MetS

The criteria of HTN and MetS were in accordance with the description in our previous study [23, 28]. Briefly, the definition of HTN was SBP at least 140 mmHg, DBP at least 90 mmHg, or current anti-hypertensive treatment, consistent with the JNC-7 criteria. Because type 2 diabetes mellitus or hyperglycemia was one criterion for MetS and all of our diabetic patients fulfilled the criteria on the basis of the updated NCEP ATP III criteria for Asian-Americans, MetS was identified as the coexistence of at least two of the criteria as follows: a waist circumference at least 90 cm (men) or at least 80 cm (women); TG of 1.7 mmol/l or more; HDL-C less than 1.03 mmol/l (men) or less than 1.30 mmol/l (women); and blood pressure of 130/85 mmHg or more or anti-hypertensive medications.

### Statistical analyses

The analyses of our data were conducted with SPSS 15.0 (SPSS Inc, Chicago, IL, USA). Normality was checked for continuous variables. Data were described as means with standard deviations or medians and inter-quartile range. Normally or non-normally distributed variables were analyzed with one-way ANOVA with LSD or Kruskal–Wallis test. Categorical variables were estimated with Chi square test across the three groups. Logistic regression was performed to evaluate differences in categorical variables while controlling for sex and/or age [10–12]. General linear model univariate was undertaken to estimated differences in quantitative variables with adjustment for sex and/or age [10–12]. Binary logistic regression was utilized to determine risk factors for HTN and MetS. *P *< 0.05 was recognized as statistically significant.

## Results

### Baseline characteristics

The baseline characteristics of the different groups are displayed in Table 1. Although neither the type 1 diabetics nor the non-ketotic type 2 diabetics were observed with a statistical difference in gender distribution, the ketosis-onset diabetic patients showed a male preponderance after controlling for age. In addition, after correction for sex and/or age, age, SBP, DBP, BMI, WHR, FPG, TC, TG, HDL-C, LDL-C, HOMA2-%S, HOMA2-IR, SUA, eGFR and CRP displayed significant differences across the three diabetic groups (all *P* < 0.05).

**Table 1:** Characteristics of the newly diagnosed diabetic participants

Variables	Type 1 diabetes (n = 83)	Ketosis-onset diabetes (n = 279)	Non-ketotic type 2 diabetes (n = 372)	*P*- value	‡*P*- value
Age (years)	44 ± 21	50 ± 15	55 ± 14	< 0.001	< 0.001
Male (n, %)	47 (56.6%)	194 (69.5%)	223 (59.9%)	0.018	0.018
Smoking (n, %)	19 (22.9%)	106 (38.0%)	122 (32.8%)	0.026	0.161
Alcohol (n, %)	9 (10.8%)	58 (20.8%)	73 (19.6%)	0.106	0.185
SBP (mmHg)	121 ± 14	125 ± 16	128 ± 17	< 0.001	0.038
DBP (mmHg)	76 ± 9	80 ± 10	81 ± 10	< 0.001	< 0.001
BMI (kg/m2)	21.6 ± 4	24.4 ± 3.7	24.8 ± 3.4	< 0.001	< 0.001
WHR	0.86 ± 0.07	0.91 ± 0.06	0.91 ± 0.06	< 0.001	< 0.001
FPG (mmol/l)^a^	7.7 (5.6–11.1)	9.4 (7.2–12.1)	7.8 (6.5–10)	< 0.001	< 0.001
2-hPPG (mmol/l)^a^	13.1 (9.8–17.7)	15.4 (12.2–19.3)	13.8 (10.7–17.3)	< 0.001	< 0.001
HbA1C (%)	11.11 ± 3.32	11.56 ± 2.51	10.03 ± 2.68	< 0.001	< 0.001
FCP (ng/ml)^a^	0.57 (0.27–1.19)	1.08 (0.5–1.83)	1.93 (1.27–2.85)	< 0.001	< 0.001
2-h PCP (ng/ml)^a^	1.26 (0.59–2.46)	2.04 (0.99–3.63)	4.31 (2.66–5.73)	< 0.001	< 0.001
HOMA2- %S	184.9 (86.6–344.43)	97.8 (55–193.2)	61.1 (41.53–90.85)	< 0.001	< 0.001
HOMA2-IR	0.55 (0.3–1.15)	1 (0.5–1.8)	1.6 (1.1–2.4)	< 0.001	< 0.001
TC (mmol/l)	4.46 ± 1	4.75 ± 1.17	4.75 ± 1.17	0.101	0.029
TG (mmol/l)^a^	1.04 (0.7–1.53)	1.3 (0.9–2.1)	1.5 (1.1–2.2)	< 0.001	0.010
HDL-C (mmol/l)	1.24 ± 0.36	1.07 ± 0.34	1.10 ± 0.28	< 0.001	< 0.001
LDL-C (mmol/l)	2.83 ± 0.89	3.12 ± 0.95	3.19 ± 1.00	0.010	0.002
ALT (U/l)^a^	20 (13–29)	22 (14–37)	22 (15–39)	0.380	0.534
SCr (μmol/l)^a^	61 (50–75)	66 (55–76)	66 (54–78)	0.096	0.924
SUA (μmol/l)^a^	264 (226–319)	279 (220–351)	311 (251–373)	< 0.001	< 0.001
UAE (mg/24 h)^a^	8.0 (5.2–12.5)	9.4 (6.4–20)	10.2 (6.5–19.6)	0.006	0.644
eGFR (ml/min/1.73 m^2^)^a^	123 (100–163)	121 (101–145)	115 (95–135)	0.002	0.003
CRP (mg/l)^a^	0.57 (0.2–1.63)	1.1 (0.49–3.48)	1.26 (0.53–2.96)	< 0.001	< 0.001

Data are presented as means and standard deviations, or medians with interquartile range, or percentages

*P* value: crude *P*-values

‡*P*-value: ‡*P*-values adjusted for age and gender

^a^Non-normal distribution of quantitative variables

### Comparison of HTN and MetS across the three groups

**Fig. 1:**
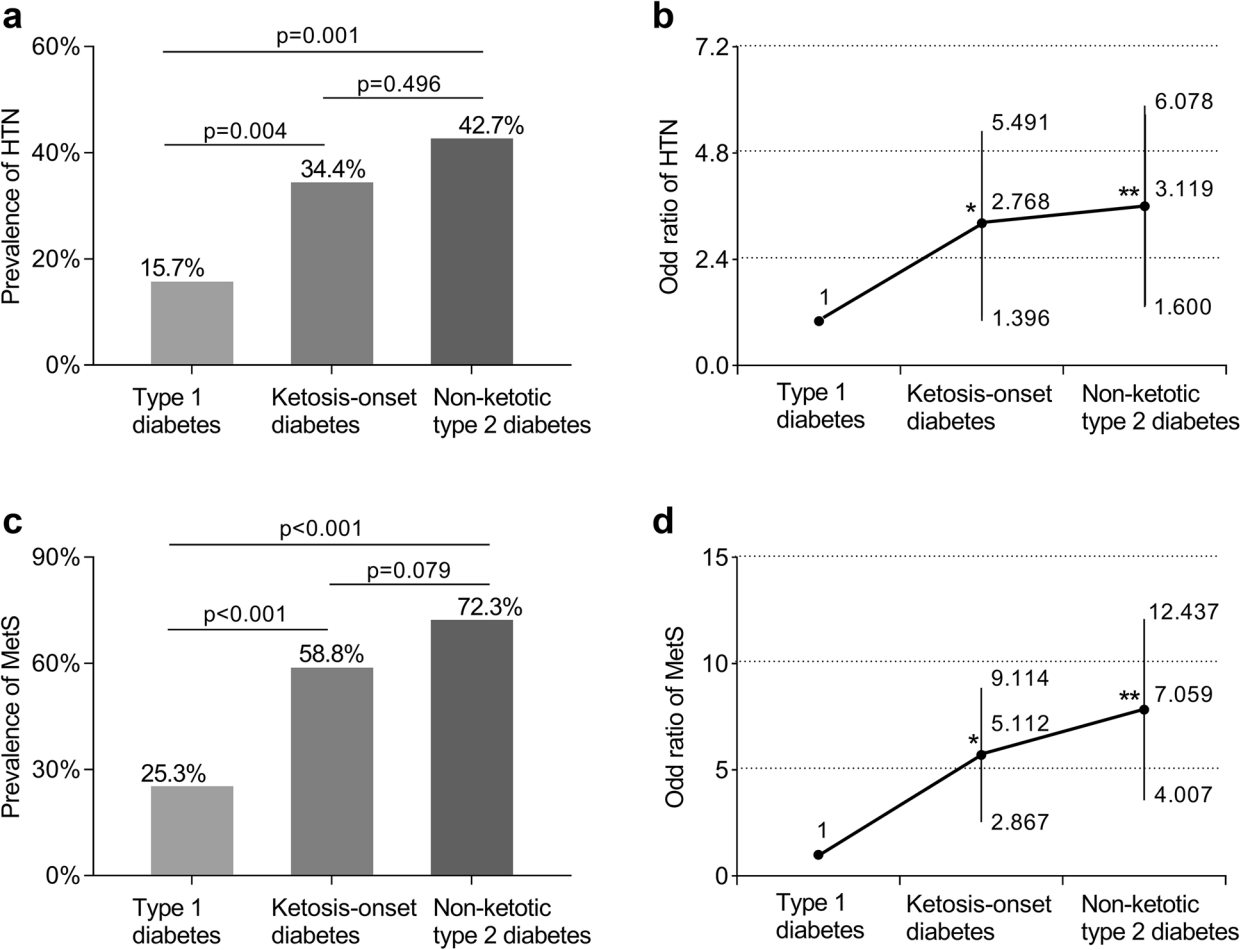
Comparison of HTN and MetS in patients with diabetes after correction for gender and/or age. **a** Comparison of the frequency of HTN across type 1, ketosis-onset and non-ketotic type 2 diabetics. **b** Odds ratio with 95% confidence interval of HTN for the ketosis-onset and the non-ketotic type 2 diabetics in contrast to the type 1 diabetic subjects. In contrast to the type 1 diabetes, where *P = 0.004 and **P = 0.001. **c** Comparison of the frequency of MetS across type 1, ketosis-onset and non-ketotic type 2 diabetics. **d** Odds ratio with 95% confidence interval of MetS for the ketosis-onset and the non-ketotic type 2 diabetics in contrast to the type 1 diabetic subjects. In contrast to the type 1 diabetes, where *P < 0.001 and **P < 0.001. *HTN* hypertension, *MetS* metabolic syndrome

As apparent from Fig. 1a, the frequency of HTN in the ketosis-onset diabetics (34.4%) was in excess of that observed in the type 1 diabetics (15.7%, *P *= 0.004), without remarkable difference compared to that in the type 2 diabetics (42.7%, *P *= 0.496), while adjusting for gender and age variables. The OR (95% CI) for HTN was higher in the ketosis-onset diabetic subjects than in those with type 1 diabetes but similar at 2.768 (95% CI 1.396–5.491) in the ketosis-onset diabetic subjects and at 3.119 (95% CI 1.600–6.078) in the non-ketotic type 2 diabetic patients while correcting for gender and age (Fig. 1b).

Likewise, MetS remained more prevalent in patients with ketosis-onset diabetic group (58.8%) than in those with type 1 diabetes (25.3%, *P *< 0.001). Nonetheless, there was no evidence that the ketosis-onset diabetic and the type 2 diabetic group (72.3%) differed in the prevalence of MetS after adjustment for gender and age (*P *= 0.079) (Fig. 1c). Referring to type 1 diabetic subjects, the OR (95% CI) for MetS were as follows: for the ketosis-onset diabetic patients 5.112 (95% CI 2.867–9.114) (*P *< 0.001) and for the type 2 diabetic patients 7.059 (95% CI 4.007–12.437) (*P *< 0.001) (Fig. 1d). No evidence was recorded that ketosis-onset and non-ketotic type 2 diabetics differed concerning the OR for MetS, despite distinction between ketosis-onset diabetics and those in type 1 diabetics.

### Comparison of HTN and MetS subdivided by gender and age in the three groups

**Fig. 2:**
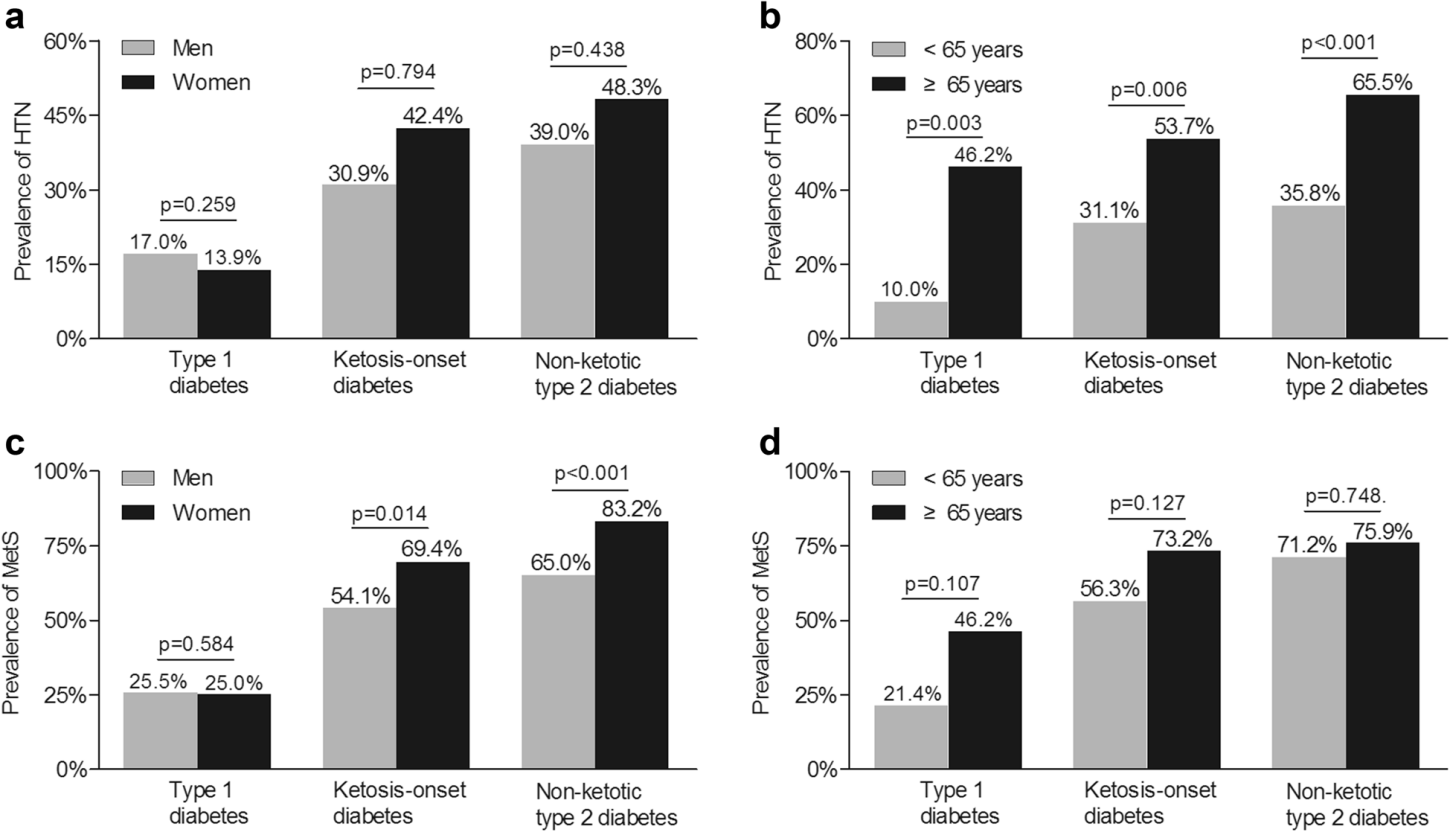
Comparison of gender- and age-stratified HTN and MetS across the three diabetic groups. **a** The gender-stratified prevalence of HTN in the three diabetic groups with adjustment for age. **b** The age-stratified prevalence of HTN in the three diabetic groups with adjustment for gender. **c** The gender-stratified prevalence of MetS in the three diabetic groups while controlling for age. **d** The age-stratified prevalence of MetS in the three diabetic groups while controlling for gender. HTN, hypertension; MetS, metabolic syndrome

Analyses of gender- and age-stratified HTN and MetS in every diabetic group are shown in Fig. 2. No association was noted between gender and the prevalence of HTN in three groups (Fig. 2a). However, the frequency of HTN in the subjects ≥ 65 years of age was higher than in the subjects < 65 years of age in all groups (Fig. 2b). On the contrary, MetS was detected in more females with ketosis-onset and non-ketotic type 2 diabetes, without the gender-linked difference in the type 1 diabetes (Fig. 2c). Differentiating from the prevalence of HTN, there was no age-associated increase in the frequency of MetS in the respective group (Fig. 2d).

### Analyses of risk factors for HTN and MetS in each diabetic group

Binary logistic regression analyses with respect to risk factors for HTN are performed in Table 2. In all diabetic patients, age was associated with risk for HTN. Additionally, in the type 1 diabetes, WHR and LDL-C were also in close relation to the presence of HTN. On the contrary, in the non-ketotic type 2 diabetes, BMI, LDL-C, and SUA were independent risk factors for HTN. Of note, BMI and SUA were also linked to risk for HTN in the ketosis-onset diabetics, which were almost identical with those in the non-ketotic type 2 diabetics.

**Table 2:** Risk factors of HTN in each diabetic group

Group	Variables	OR	95% CI	*P*-value
Type 1 diabetes	Age	1.079	1.044–1.116	< 0.001
	WHR	1.775	1.116–2.821	0.015
	LDL-C	2.241	1.379–3.642	0.001
Ketosis-onset diabetes	Age	1.082	1.045–1.222	< 0.001
	BMI	1.268	1.086–1.480	0.003
	SUA	1.004	1.000–1.009	0.050
Non-ketotic type 2 diabetes	Age	1.082	1.054–1.109	< 0.001
	BMI	1.153	1.051–1.266	0.003
	LDL-C	1.533	1.129–2.080	0.006
	SUA	1.004	1.001–1.008	0.024

Adjusted for age, sex, smoking, alcohol drinking, BMI, ALT, TC, LDL-C, CRP, HbA1C, FPG, 2 h PPG, FCP, 2 h PCP, Cr, SUA, UAE, and eGFR

Table 3 provides the risk factors for MetS by binary logistic regression analyses in diabetic patients. Sex and BMI were associated with risk for MetS in all type of diabetes. Similar to HTN, the risk factors for MetS including SUA and FCP in the ketosis-onset diabetics also occurred in the non-ketotic type 2 diabetic subjects. In addition, age and HbA1C were also correlated with the risk of MetS in the non-ketotic type 2 diabetes. However, besides sex and BMI, the risk factors related to MetS in the type 1 diabetes were different as follows: age, LDL-C, and CRP.

**Table 3:** Risk factors of MetS in each diabetic group

Group	Variables	OR	95% CI	*P*-value
Type 1 diabetes	Age	1.073	1.029–1.119	0.001
	Sex (reference: men)	3.245	1.058–9.951	0.040
	BMI	1.675	1.345–2.086	< 0.001
	LDL-C	3.043	1.590–5.823	0.001
	CRP	1.038	1.002–1.075	0.038
Ketosis-onset diabetes	Sex (reference: men)	3.247	1.738–6.067	< 0.001
	BMI	1.503	1.337–1.690	< 0.001
	SUA	1.003	1.000–1.007	0.047
	FCP	1.44	1.017–2.037	0.040
Non-ketotic type 2 diabetes	Age	1.036	1.007–1.066	0.014
	Sex (reference: men)	3.615	1.657–7.890	0.001
	BMI	1.377	1.196–1.587	< 0.001
	SUA	1.009	1.004–1.015	0.001
	FCP	2.192	1.394–3.447	0.001
	HbA1C	1.21	1.049–1.396	0.009

Adjusted for age, sex, smoking, alcohol drinking, BMI, ALT, TC, LDL-C, CRP, HbA1C, FPG, 2 h PPG, FCP, 2 h PCP, Cr, SUA, UAE, and eGFR

## Discussion

According to our surveys, HTN and MetS were frequent findings in ketosis-onset diabetes. Ketosis-onset diabetes was comparable to non-ketotic type 2 diabetes but exceeded type 1 diabetes regarding the prevalence and clinical features of HTN and MetS, supporting the categorization of ketosis-onset diabetes.

Our findings showed that ketosis-onset diabetes presented a higher predominance in men, which was reported 1:5 to 6:1 male to female ratios in the previous observations, whereas type 1 and non-ketotic type 2 diabetes were more females [30, 31]. Men accumulated more adipose tissue in the visceral area and higher levels of androgen related with insulin-resistance and were more susceptible to glucotoxicity or glucolipotoxicity triggering unprovoked ketosis [30]. FPG levels ranged from 6.2 to 11.5 mmol/l and 2 h PPG levels ranged from 18.3 to 20.4 mmol/l in ketosis-onset diabetes reported in the past studies [21, 31]. Likewise, our findings showed that patients with ketosis-onset diabetes had the highest FPG and 2 h PPG compared with type 1 or type 2 diabetic controls. Beyond other types of diabetes, high levels of plasma glucose in ketosis-onset diabetes exhibited the acute blunted insulin secretion and an initial reduction in glucose disposal at the episode of ketoacidosis [2, 32]. Moreover, ketosis-onset diabetes was observed to be related with middle age at diagnosis and obesity, which were different from type 1 diabetes. HbA1c levels in ketosis-onset diabetes were higher than in type 2 diabetes, while remained similar in contrast to those in type 1 diabetes. According to the past surveys, HbA1c levels were from 5.7 to 11.5% in ketosis-onset diabetes, from 7.8 to 10.6% in type 1 diabetes, and from 11.8 to 12.5% in type 2 diabetes, which were found no statistically difference among three groups [1, 6, 21, 33, 34].

HTN rate in the ketosis-onset diabetes was distinct from that in the type 1 diabetes, whereas showed no difference between ketosis-onset diabetes and non-ketotic type 2 diabetes in our current study. The proportion of non-ketotic type 2 diabetics with HTN was 42.7% in our present study, close to the 50% reported in the previous study [15]. By comparison, our research reflected that 15.7% of type 1 diabetes was hypertensive, and approximately 11–59% of type 1 diabetic patients had HTN based on different diagnostic criteria by previous investigators [17, 35].

The frequency of HTN in ketosis-onset diabetes reported herein was 34.4%, near to that of non-ketotic type 2 diabetes. There were only two previous studies referring to the prevalence of HTN in small samples of ketosis-onset diabetes without comparison with type 1 and non-ketotic type 2 diabetes [33, 36]. Goodstein et al. [36] reported 25 HTN patients in 33 ketosis-onset diabetes in the veteran population. Pinero-Pilona et al. [33] also undertook a small observational study, in which the prevalence of HTN was 29.7% in 37 ketosis-onset diabetic patients at follow-up. In our study, 279 ketosis-onset diabetics were related to 2.8-fold risk of HTN compared with the type 1 diabetic subjects, whereas with undifferentiated OR compared with the type 2 non-ketotic diabetics. It assumed that ketosis-onset diabetes might correlate with insulin resistance and sympathetic activation, which always occurred in non-ketotic type 2 diabetes, progressing to develop HTN and cardiac abnormalities such as diastolic dysfunction, macroangiopathy and left ventricular hypertrophy [26, 37].

It was described that high levels of BMI and SUA, which was the characteristics of HTN in type 2 diabetes, was also linked to HTN in ketosis-onset diabetes. In contrast, LDL-C and WHR were at risk for HTN independently in type 1 diabetes, which was different from those of ketosis-onset diabetes. Therefore, risk factors of HTN in the ketosis-onset diabetes were close to those of the non-ketotic type 2 diabetes instead of type 1 diabetes.

On the other hand, MetS was more frequent in ketosis-onset diabetics and non-ketotic type 2 diabetics than in type 1 diabetics. The prevalence of MetS in type 2 diabetic population reached 65–75.6% [24, 38]. In our previous observations, approximately 70% of Chinese type 2 diabetic outpatients had features of the MetS [23]. Nonetheless, Hawa et al. [22] found that MetS was not a characteristic of autoimmune type 1 diabetes, which had comparatively lower prevalence despite the study population and the diagnostic criteria. MetS was detected in 58.8% of the patients with ketosis-onset diabetes in our research, higher than in the type 1 diabetes (25.3%). Furthermore, Otiniano et al. [39] reported that 74 ketosis-prone diabetic patients with MetS were inclined to have traits to type 2 diabetes.

Despite lacking direct research for MetS in ketosis-onset diabetes, there were a few studies observed that individual components of MetS were similar in patients with ketosis-onset diabetes and non-ketotic type 2 diabetes, but unlike those in type 1 diabetes [1, 40]. As one of the key components of MetS, the average level of TG in ketosis-onset diabetes approached that of non-ketotic type 2 diabetes, in striking contrast with type 1 diabetes [18, 41]. Moreover, TC, HDL-C, and LDL-C bore similarity between ketosis-onset diabetes and type 2 diabetes, in consistence with the past studies [7, 20]. Additionally, the WHR values were found undifferentiated between the ketosis-onset and non-ketotic type 2 diabetes in the present research, which was in accordance with a previous study [18, 41]. However, higher values of WHR were detected in ketosis-onset diabetics than in type 1 diabetics. Goodstein et al. [36] showed that ketosis-onset diabetic patients accompanied by three or more metabolic-related risk variables of type 2 diabetes comprising obesity, dyslipidemia, and HTN, predicting beta-cell recovery. Bhalla et al. [34] emphasized that ketosis-onset diabetes displayed a similar level of insulin resistance compared with type 2 diabetes. Lontchi-Yimagou et al. [19] showed that HOMA-IR was higher in type 2 diabetes than in ketosis-onset diabetes. Of note, the insulin secretion evaluated by FCP and 2-h PCP and insulin resistance quantified by HOMA2-IR were intermediate between type 1 diabetics and non-ketotic type 2 diabetics in our research, which may occur due to remission duration after initial metabolic disturbance. As a result, ketosis-onset diabetic individuals had a higher frequency of MetS, approaching those of non-ketotic type 2 diabetes instead of type 1 diabetes subjects. Moreover, identically, risk factors of MetS in the ketosis-onset diabetes were almost in conformity with those in the non-ketotic type 2 diabetes, rather than those in the type 1 diabetes, which indirectly supported that ketosis-onset diabetes were proposed as a subtype of type 2 diabetes.

Our results had clinical and therapeutic inferences for ketosis-onset diabetes. Firstly, the prevalence of HTN and MetS were similar in participants with ketosis-onset diabetes and type 2 diabetes but was higher in both groups than in type 1 diabetic group, which elucidated that ketosis-onset diabetes tended to be classified into type 2 diabetes. Control of diet and exercise, and insulin sensitizers treatment to minimize insulin resistance and to mitigate the risk of relapse might be suitable for ketosis-onset diabetes after the initial hyperglycemia crisis. Secondly, we observed that ketosis-onset diabetes was associated with similar metabolic and hypertensive risk as type 2 diabetes. Senility, overweight, hyperuricemia, and hyperinsulinemia were the comorbidity with HTN or MetS in ketosis-onset diabetes. Therefore, early screening and stringent control of HTN, dyslipidemia, overweight, and other modifiable risk factors in MetS was necessary for estimation and reduction of cardiovascular risk in ketosis-onset diabetes.

Limitations should also be acknowledged. Firstly, potential confounding factors could affect the results, due to the single-center and observational study. However, we controlled these confounding factors as much as possible in the present analyses. Secondly, the study did not assess other diagnostic criteria of MetS. Although disparity was found in the diagnosis of criteria, the NCEP ATP III criteria were simple and appropriate to apply in Asian patients compared with the IDF criteria or the WHO criteria [22].

## Conclusions

In summary, both HTN and MetS were frequent findings in ketosis-onset diabetic patients. The prevalence of HTN and MetS in the ketosis-onset diabetics were magnificently greater than in the type 1 diabetics but showed no difference in comparison to the type 2 diabetics. The clinical features and risk factors of HTN and MetS in the ketosis-onset diabetes were close to those in the non-ketotic type 2 diabetes but distinguished from those in the type 1 diabetes. We can put forward that ketosis-onset diabetes should be categorized into type 2 diabetes, not idiopathic type 1 diabetes.

## Abbreviations

HTN: hypertension
MetS: metabolic syndrome
GAD: glutamic acid decarboxylase
IA-2: tyrosine phosphatase-like islet antigen 2
BMI: body mass index
WHR: waist-to-hip ratio
SBP: systolic blood pressure
DBP: diastolic blood pressure
HbA1C: glycated hemoglobin A1C
FPG: fasting plasma glucose
2-h PPG: 2-h postprandial plasma glucose
HOMA2- %S: HOMA of insulin sensitivity
HOMA2-IR: HOMA of insulin resistance
FCP: fasting C-peptide
2-h PCP: 2-h postprandial C-peptide
TC: total cholesterol
TG: total triglycerides
HDL-C: high-density lipoprotein cholesterol
LDL-C: low-density lipoprotein cholesterol
SUA: serum uric acid
ALT: alanine transaminase
SCr: serum creatinine
UAE: urinary albumin excretion
CRP: C-reactive protein
eGFR: estimated glomerular filtration rate
